# Blood–Brain Barrier Disruption Mediated by FFA1 Receptor—Evidence Using Miniscope

**DOI:** 10.3390/ijms23042258

**Published:** 2022-02-18

**Authors:** Kristen L. Lindenau, Jeffrey L. Barr, Christopher R. Higgins, Kevin T. Sporici, Eugen Brailoiu, Gabriela C. Brailoiu

**Affiliations:** 1 Department of Pharmaceutical Sciences, Jefferson College of Pharmacy, Thomas Jefferson University, Philadelphia, PA 19107, USA; kristen.lindenau@gmail.com (K.L.L.); christopher.higgins@students.jefferson.edu (C.R.H.); kevintudor@gmail.com (K.T.S.); 2 Department of Neural Sciences and Center for Substance Abuse Research, Lewis Katz School of Medicine, Temple University, Philadelphia, PA 19140, USA; jeffrey.barr@sanfordhealth.org

**Keywords:** BBB, brain microvascular endothelial cells, ECIS, omega-3 fatty acids, n-3 PUFAs

## Abstract

Omega-3 polyunsaturated fatty acids (n-3 PUFAs), obtained from diet and dietary supplements, have been tested in clinical trials for the prevention or treatment of several diseases. n-3 PUFAs exert their effects by activation of free fatty acid (FFA) receptors. FFA1 receptor, expressed in the pancreas and brain, is activated by medium- to long-chain fatty acids. Despite some beneficial effects on cognition, the effects of n-3 PUFAs on the blood–brain barrier (BBB) are not clearly understood. We examined the effects of FFA1 activation on BBB permeability in vitro, using rat brain microvascular endothelial cells (RBMVEC), and in vivo, by assessing Evans Blue extravasation and by performing live imaging of brain microcirculation in adult rats. AMG837, a synthetic FFA1 agonist, produced a dose-dependent decrease in RBMVEC monolayer resistance assessed with Electric Cell–Substrate Impedance Sensing (ECIS); the effect was attenuated by the FFA1 antagonist, GW1100. Immunofluorescence studies revealed that AMG837 produced a disruption in tight and adherens junction proteins. AMG837 increased Evans Blue content in the rat brain in a dose-dependent manner. Live imaging studies of rat brain microcirculation with miniaturized fluorescence microscopy (miniscope) showed that AMG837 increased extravasation of sodium fluorescein. Taken together, our results demonstrate that FFA1 receptor activation reduced RBMVEC barrier function and produced a transient increase in BBB permeability.

## 1. Introduction

The blood–brain barrier (BBB) is a functional and structural barrier essential for homeostasis to allow for proper neural function and for protecting the brain against circulating toxins and pathogens [1]. BBB is comprised of endothelial cells, astrocytes and pericytes [1,2]. The microvascular endothelial cells that line the walls of capillaries in the central nervous system form a tight monolayer with low paracellular permeability [2]. A core function of brain microvascular endothelial cells is to limit passive diffusion of potentially harmful substances across the BBB and preventing them from entering the brain. 

Omega-3 polyunsaturated fatty acids (n-3 PUFAs), such as docosahexaenoic acid (DHA) and eicosapentaenoic acid (EPA) obtained from diet or dietary supplements [3,4], have been reported to have beneficial neurological effects by improving cognitive performance [5,6,7,8] in neurodegenerative disorders and depression [9,10]. Recently, DHA and EPA were reported to promote BBB integrity in a sample of healthy elderly (mean age 76 yrs) human subjects [11]. Animal studies indicate that a diet rich in n-3 PUFAs reduces BBB disruption associated with aging [12]. However, in rat models of ischemia–reperfusion injury, both beneficial [13] and detrimental [14] effects on BBB permeability were reported. When administrated after reperfusion, DHA was shown to increase BBB permeability and cause brain edema [14]. Other studies showed that DHA confers long-term protection against ischemic brain damage through multiple mechanisms, including suppression of inflammatory responses, decrease in oxidative stress and stimulation of angiogenesis and neurogenesis [15,16]. 

DHA is an endogenous agonist of free fatty acid receptor 1 (FFA1) [17], previously named GPR40 [18]. FFA1 receptor is predominantly expressed in pancreatic β cells where it plays an important role in glucose homeostasis [19] and has emerged as a potential target for the treatment of type II diabetes [20,21]. FFA1 is also expressed in various brain regions where is involved in physiological processes such as neurogenesis and neurodevelopment and in pathophysiological conditions such as apoptosis, inflammatory pain, Alzheimer’s disease and Parkinson’s disease [22,23,24,25]. We recently reported a potential cardioprotective effect of FFA1 receptor activation in brainstem neurons of nucleus ambiguus that control the cardiac parasympathetic tone [26].

Given the inconclusive reports of both beneficial and negative effects of DHA on the BBB, further research is needed to establish the impact of FFA1 agonists on BBB function. The present study utilized multiple models and endpoints to address this question. We examined the effect of FFA1 activation produced by acute treatment with AMG837, a synthetic FFA1 agonist [27], on BBB permeability using the in vitro model of rat brain microvascular endothelial cells by evaluating monolayer electrical resistance and tight and adherens junction proteins. Complementary in vivo studies in rats directly assessed the effects of AMG837 on BBB permeability using intracranial microscopy and extravasation of dye into brain tissue. Together, our results demonstrate that AMG837, acting through activation of FFA1 receptor, transiently disrupts BBB function.

## 2. Results

### 2.1. FFA1 Activation Produces a Transient Decrease in RBMVEC Monolayer Resistance 

**Figure 1:**
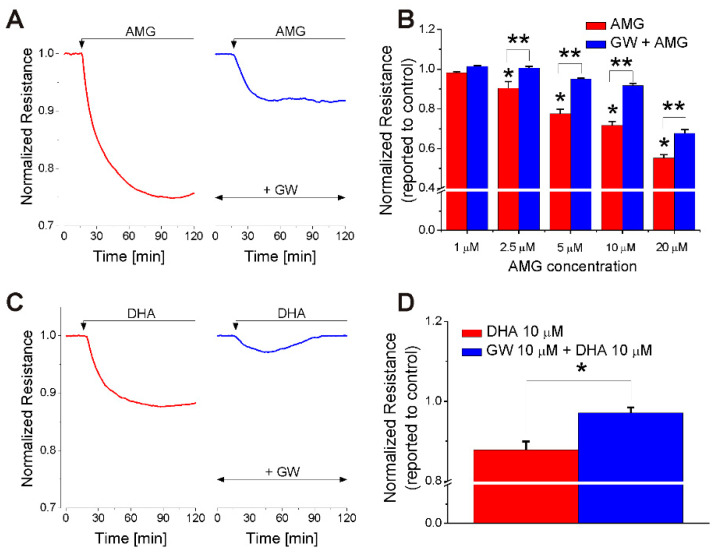
FFA1 mediated an acute decrease in the normalized resistance of RBMVEC monolayer assessed with Electric Cell–Substrate Impedance Sensing (ECIS). (**A**) Representative example of a decrease in normalized resistance produced by AMG837 (10 μM); pretreatment with the FFA1 antagonist GW1100 (10 μM) reduced the effect of AMG837. (**B**) Comparison of the effect of AMG (1–20 μM) on RBMVEC resistance in the absence and presence of GW1100 (10 μM); AMG837 (2.5, 5, 10, 20 μM) produced a dose-dependent decrease in the normalized resistance of RMBVEC monolayer. *p* < 0.05 as compared to the effect of AMG837 (1 μM) (*) or to the effect of the same concentration of AMG837 in the presence of GW1100 (10 μM) (**). (**C**) Representative example of a decrease in normalized resistance produced by DHA (10 μM); pretreatment with GW1100 (10 μM) markedly decreased the effect of DHA. (**D**) Comparison of the reduction in normalized resistance produced by DHA (10 μM) in the absence and presence of GW1100 (10 μM). * *p* < 0.05.

Impedance measurements via Electric Cell–Substrate Impedance Sensing (ECIS) were carried out in RBMVEC monolayers grown on gold electrodes of 8W10E+ arrays. AMG837 (10 μM), a synthetic FFA1 agonist [27] produced a robust reduction in normalized resistance (Figure 1A). Pretreatment with GW1100, a FFA1 antagonist (10 μM, 15 min) [28,29], produced an important attenuation of the amplitude and duration of the response to AMG837. Representative examples of AMG-induced reduction in normalized resistance in the absence and presence of GW1100 over a 2 h period are shown in Figure 1A. AMG837 (1, 2.5, 5, 10, and 20 μM) caused a concentration-dependent reduction in the normalized electrical resistance of RBMVEC monolayers to 0.98 ± 0.004, 0.90 ± 0.031, 0.77 ± 0.021, 0.71 ± 0.012, and 0.55 ± 0.015, respectively (n = 8–10/concentration tested) (Figure 1B; red bars). In cells pretreated with GW1100, a FFA1 antagonist (10 μM, 15 min) [28,29], a significant attenuation in the response to AMG837 (2.5–20 μM) was noted (Figure 1B; blue bars). These results indicate that activation of FFA1 reduces the electrical resistance of the RBMVEC monolayers in a concentration-dependent manner. DHA (10 μM) reduced the normalized resistance of RBMVEC monolayer to 0.87 ± 0.021; GW1100 (10 μM) markedly decreased the effect of DHA to 0.97 ± 0.011 (Figure 1C,D). 

**Figure 2:**
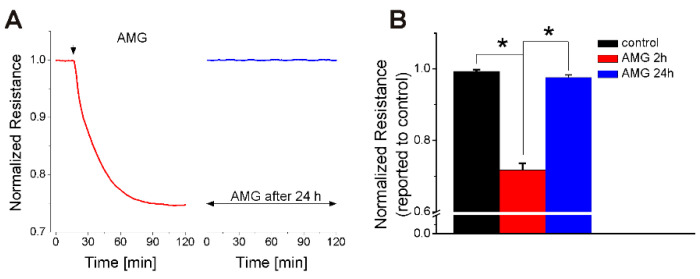
FFA1-mediated effect on normalized RBMVEC resistance returned to basal levels within 24 h after treatment with AMG837. (**A**) Representative example of normalized resistance recorded with ECIS during treatment with AMG837 (10 μM) in the first 2 and after 24 h. (**B**) Comparison of the effect of AMG837 on normalized resistance at 2 and 24 h. * *p* < 0.05.

Normalized RBMVEC resistance returned to basal levels within 24 h after treatment with AMG837 (Figure 2A,B). Figure 2A shows the effect of AMG837 on resistance in the left panel (first 90 min after application) and the same measurement made 24 h after AMG837. 

### 2.2. AMG837 Alters RBMVEC Tight and Adherens Junctions and Cytoskeleton

**Figure 3:**
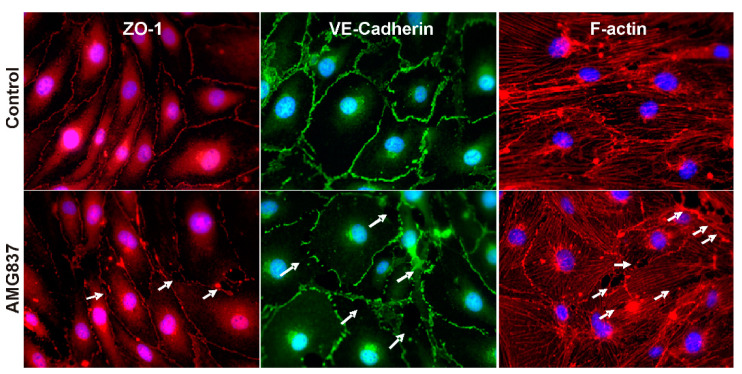
AMG37 produced a disruption in tight and adherens junction proteins and F-actin in RBMVEC. Distribution of tight junction accessory protein ZO-1, adherens junction protein VE-cadherin, and cytoskeleton component F-actin in control RBMVEC and RBMVEC treated with AMG837 (10 µM, 30 min). Nuclei are stained with DAPI. Treatment with AMG837 produced a disruption in ZO-1 and VE-cadherin, reorganization of F-actin and the formation of intercellular gaps (arrows).

Immunocytochemistry studies examined the distribution of ZO-1, a tight junction accessory protein that connects to the actin cytoskeleton, of VE-cadherin, an adherens junction protein, and of F-actin cytoskeleton in RBMVEC before and after treatment with AMG837. In control RBMVEC, ZO-1 and VE-cadherin proteins were visualized as well defined, continuous structures bordering and connecting the cells and F-actin cytoskeleton as intracellular filaments throughout the cells (Figure 3 top panels). Treatment of RBMVEC with AMG837 (10 μM, 30 min) produced a disruption in ZO-1 and VE-cadherin proteins, visualized as discontinuous structures between the cells and a reorganization of F-actin stress fibers leading to formation of intercellular gaps, indicated with arrows (Figure 3 bottom panels). 

### 2.3. AMG837 Increases Brain Evans Blue Extravasation 

**Figure 4:**
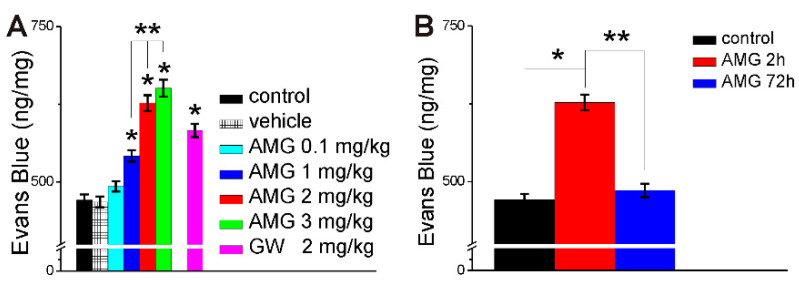
AMG837 increased Evans Blue content in the brain. (**A**) Systemic administration of AMG837 (0.1–3 mg/kg, i.v.) increased Evans Blue content in the brain, determined at 2 h after treatment, in a dose-dependent manner, indicating an increase in BBB permeability. GW1100 (2 mg/kg, i.v.) also increased Evans Blue. *P* < 0.05 as compared to control or vehicle-injected rats (*) or compared with AMG 837 (1–3 mg/kg) (**). (**B**) Comparison of Evans Blue concentration in the rat brain in control rats, and 2 and 72 h after AMG (2 mg/kg, i.v.). *p* < 0.05 as compared to control (*) or to effect of AMG at 72 h (**).

Rats were injected i.v. with Evans Blue 150 min prior to collection of brains. In otherwise naive rats, the brain concentration of Evans Blue was 471 ± 9.2 ng/mg, which was similar to that in rats injected with vehicle (DMSO, 0.1% *v*/*v*, 250 µL) 120 min prior to brain collection (468 ± 8.7 ng/mg). Injection of AMG837 (0.1, 1, 2, or 3 mg/kg, i.v) increased the brain concentration of Evans Blue at 2 h to 493 ± 8.4 ng/mg, 542 ± 9.5 ng/mg; 627 ± 12.7 ng/mg and 651± 13.4 ng/mg, respectively (n = 6 rats/dose) (Figure 4A). In other series of experiments, i.v. administration of GW1100 (2 mg/kg, 250 µL), a FFA1 antagonist, increased brain Evans Blue concentration to 583 ± 10.6 ng/mg (Figure 4A.). Evans Blue concentration in the brain at 72 h after AMG837 (2 mg/kg) was reduced to 486 ± 10.8 ng/mg, similarly to control (471± 9.2 ng/mg) as compared to 627 ± 12.7 ng/mg at 2 h (Figure 4B).

### 2.4. AMG837 Increases Sodium Fluorescein Extravasation Visualized with Miniscope 

**Figure 5:**
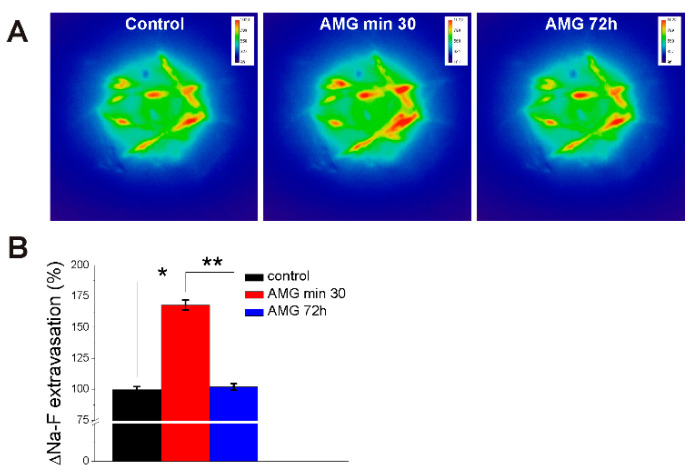
AMG837 increases sodium fluorescein (Na-F) extravasation in brain microvessels visualized with miniscope. (**A**) Pseudocolor images of Na-F fluorescence in rat prefrontal cortex before (control), 30 min and 72 h after injection of AMG837. (**B**) Comparison of Na-F extravasation (%) in brain microvessels in control and AMG (2 mg/kg)-treated rats, at 30 min and 72 h, determined by averaging the fluorescence intensity for 10 ROIs in the vicinity of microvessels 15 min after i.v. administration of Na-F. AMG837 increased Na-F extravasation at 30 min after injection. *p* < 0.05 as compared to control (*) or to effect of AMG at 72 h (**).

Brain microcirculation in the prefrontal cortex was visualized with the miniscope following sodium fluorescein administration in awake, freely-moving rats, as we recently reported [30,31] before and after AMG837 injection (Figure 5A). AMG837 (2 mg/kg, i.v.) increased sodium fluorescein extravasation at 30 min to 168 ± 4.1%, as compared to 99.8 ± 2.4% in control rats (n = 6). At 72 h post-AMG837 injection, the sodium fluorescein extravasation returned to values similar to control (102 ± 2.7%; n = 6) (Figure 5B) as noted in ex vivo experiments assessing Evans Blue concentration in the brain at 72 h after AMG837 (Figure 4B).

## 3. Discussion

Free fatty acid receptor 1 (FFA1) is a G protein-coupled receptor activated by medium and long chain fatty acids, expressed mainly in pancreas and brain [17]. In the central nervous system, FFA1 receptor was identified in various brain regions such as medulla oblongata, substantia nigra, cerebellum, hippocampus, olfactory bulb, striatum, hypothalamus, and cerebral cortex [22,25]; it was also present at high levels in the brain microvessels [32]. Docosahexaenoic acid (DHA), the endogenous ligand for FFA1, is the most predominant omega-3 polyunsaturated fatty acid (n-3 PUFA) in the brain, representing 40% of total brain PUFAs [33]. In the nervous system, FFA1 activation has been implicated in neuronal differentiation and plasticity [34], emotional behavior [35,36], and pain modulation [23]. A synthetic FFA1/GPR40 agonist, GW9508, was shown to improve the cognitive impairment in a mouse model of Alzheimer’s disease [37,38,39].

There is limited information regarding the effect of n-3 PUFAs on blood–brain barrier (BBB) function. A n-3 PUFA-enriched diet of pregnant rats had protective effects and preserved BBB structure and function in neonate rats subjected to hypoxic–ischemic brain injury [13]. Other studies reported long-term benefits on preserving BBB integrity after n-3PUFAs/DHA administration for a few days [15] to a few months [12,16]. In a mouse model of Alzheimer’s disease, 4 weeks treatment with fish oil improved the BBB disruption associated with the progression of the disease [40]. However, when DHA was administered 60 min after reperfusion in a rat model of ischemia–reperfusion, increased BBB permeability and cerebral infarction size were observed [14]. In a rat model of traumatic brain injury, DHA administered 30 min after the injury reduced BBB permeability and cerebral edema [41]. These reports indicating both beneficial and detrimental effects of n-3PUFAs/DHA on the BBB used different experimental models and long-term treatment.

We examined the effect of FFA1 receptor activation in rat brain microvascular endothelial cells (RBMVEC), a key component of the BBB. We assessed the effect of acute treatment with AMG837, a FFA1 receptor-selective agonist [27], on RBMVEC permeability in vitro by monitoring the electrical resistance of the RBMVEC monolayer using Electric Cell–Substrate Impedance Sensing (ECIS). ECIS is a reliable and sensitive method of assessing the barrier function in real time, based on the impedance measurements at several AC frequencies of cells grown on gold electrodes of ECIS arrays [42,43,44]. AMG837 produced a concentration-dependent reduction in normalized resistance measured at 4000 Hz, indicative of an increase in paracellular permeability. To further demonstrate that the reduction in resistance was FFA1 dependent, RBMVEC grown on ECIS arrays were pretreated with the FFA1 antagonist GW1100 before the addition of AMG837. Pretreatment with GW100 (10 μM), in a concentration similar to that used in other studies [28,45,46], reduced the effect of AMG837 on RBMVEC resistance. RBMVEC resistance returned to basal levels within 24 h after treatment with AMG837. 

We also tested the effect of DHA on normalized resistance; DHA (10 μM) reduced normalized resistance, but AMG837 was more potent. The effect of DHA was markedly reduced by GW1100. Previous studies indicate that DHA, the endogenous FFA1 agonist, binds at the orthosteric site, while AMG837 binds at an allosteric site with higher potency [27,47]. DHA (10–100 μM) has been reported to produce a dose-dependent decrease in transendothelial electrical resistance (TEER) of Caco-2 cell monolayers, an experimental model for the intestinal epithelial barrier [48,49,50]. Solid substrate-based techniques, such as ECIS, confer advantages over TEER measurements with Ag/AgCl chopstick electrodes, as they accurately detect changes in the electrical resistance caused by subtle changes in cell morphology and intercellular junctions and provide information on paracellular permeability [43,44].

Microvascular endothelial cells of the BBB are characterized by low paracellular permeability due to their tight and adherens junctions [1]. Tight junctions consist of a network of transmembrane proteins including occludin, claudin-5, and junctional adhesion molecules that connect to the actin cytoskeleton via adaptor proteins, such as ZO-1 [2]. Adherens junctions such as VE-cadherin connect to the cytoskeleton through catenin proteins. Disruption of tight/adherens junctions and the reorganization of the actin cytoskeleton are associated with barrier disruption and increased barrier permeability [44]. Results of our immunofluorescence studies indicate that treatment of RBMVEC with AMG837 produced a disruption in ZO-1 and VE-cadherin staining, increased F-actin stress fibers, and the formation of intercellular gaps. Similar changes were produced in brain microvascular endothelial cells by other GPCR agonists, such as bradykinin [51], or thrombin [44,52]. The morphological changes observed in immunocytochemistry studies are consistent with the reduction in the electrical resistance of the RBMVEC monolayer assessed by ECIS. Similarly, the decrease in TEER produced by DHA in Caco-2 cells was associated with changes in tight junctions [48,49].

We complemented our in vitro studies with in vivo experiments to investigate the effect of FFA1 activation on BBB permeability. AMG837 produced a dose-dependent increase in brain Evans Blue concentration determined after 2 h, indicating an increase in BBB permeability. When Evans Blue concentrations in the brain were determined 72 h after AMG837, the levels were similar to control rats. An intriguing finding was the fact that GW1100, a FFA1 antagonist, produced an increase in Evans Blue accumulation in the brain. This unexpected effect could be due to interaction to a different target, during systemic administration. In our recent study examining the effect of DHA on neurons of nucleus ambiguus, local administration of GW1100, by microinjection into nucleus ambiguus, prevented the effect of DHA [26]. In other studies, GW1100 administered by intrathecal administration prevented the antinociceptive effect of GW9508, a FFA1 agonist [24]. Intracerebroventricular (i.c.v) administration of GW1100 exacerbated incision-induced mechanical allodynia in mice [23],

In addition to this classical method of assessing BBB permeability with Evans Blue, we performed visualization of sodium fluorescein extravasation from brain microcirculation with miniscope, a newer, state-of-the-art technology developed to examine the in vivo neuronal activity [53]. We, recently, optimized this technique for the study of BBB permeability by assessing the fluorescent tracer extravasation at selected regions of interest (ROIs) in close proximity to the brain microvessels in real time, in awake rats [30,31]. AMG837 produced an increase in sodium fluorescein extravasation at 30 min after injection; the dye extravasation was similar to basal levels at 72 h after treatment. To our knowledge, this is the first study assessing the direct effect of FFA1 activation on BBB permeability in awake, freely-moving rats using intracranial microscopy (miniscope). Our results unravel a transient effect of FFA1 on increasing in vivo BBB permeability. The increase in BBB permeability produced by FFA1 receptor activation is significant given the fact that the endogenous agonist of the receptor, DHA is present in the diet, dietary supplements, and component of lipid-lowering drugs [3,4]. Lysophosphatidylcholine (LPC), an endogenous ligand for GPR119 receptor, has also been reported to exert some of its metabolic effects by activating FFA1/GPR40 and GPR55 [54,55,56]. Moreover, FFA1-selective agonists are still being considered as potential therapeutic agents for the treatment of metabolic disorders such as type 2 diabetes [57,58,59]. In addition, the transient increase in BBB permeability produced by FFA1 activation can facilitate access to the brain by other drugs which are administered concomitantly with DHA/n-3 PUFAs, or can be used as a potential tool to improve drug delivery to the brain [60,61]. 

Taken together, our results show that FFA1 activation produces an acute increase in BBB permeability in RBMVEC by disrupting tight/adherens junctions.

## 4. Materials and Methods

### 4.1. Chemicals and Reagents

AMG837 hemicalcium salt and docosahexaenoic acid (DHA), FFA1 agonists [27,47] were purchased from Tocris Bioscience (Bio-Techne, Minneapolis, MN, USA). GW1100, a FFA1 antagonist [28,29], was from Cayman Chemicals (Ann Arbor, MI, USA). AMG837, DHA and GW1100 were dissolved in DMSO and stored at −20 °C. Final concentrations were prepared in Hanks’ Balanced Salt Solution (HBSS) just before administration.

### 4.2. Animals

Adult male Sprague Dawley rats (Charles River Laboratories Inc., Wilmington, MA, USA) were housed on a 12 h light/dark cycle with free access to chow and water. Animal protocols were approved by the Institutional Animal Care and Use Committee of Temple University. This study followed the Animal Research: Reporting In Vivo Experiments (ARRIVE) guidelines and the National Institutes of Health guide for the Care and Use of Laboratory Animals.

### 4.3. Cell Culture

Rat brain microvascular endothelial cells (RBMVEC) from Cell Applications, Inc. (San Diego, CA, USA) were cultured in rat brain endothelial basal medium enriched with endothelial growth supplements, according to the manufacturer’s instructions at 37 °C and 5% CO_2_, as previously reported [62,63]. Cells were grown on T75 flasks coated with attachment factor (Cell Applications, Inc., San Diego, CA, USA) or were plated on round coverslips of 12 mm in diameter (immunocytochemistry studies), coated with human fibronectin (Discovery Labware, Bedford, MA, USA). For impedance measurements, cells were grown on 8W10E+ arrays (Applied Biophysics, Inc., Troy, NY, USA) coated with fibronectin.

### 4.4. Impedance Measurements

Impedance measurements were performed via the Electric Cell–Substrate Impedance Sensing (ECIS) method using a Zθ controller, a 16 W array holder station, and gold electrode arrays (8W10E+), as previously reported [63]. RBMVEC were cultured at a density of 100,000 cells/cm^2^ on 8W10E+ arrays coated with fibronectin (50 μg/mL, 200 μL/well, 30 min, 37 °C) and treated with L-cysteine (10 mM, 200 µL/well, 30 min, room temperature). Cells were grown on arrays in complete RBMVEC medium (Cell Applications) for 48–72 h in an incubator (37 °C, 5% CO_2_) and then transferred to FBS-free medium before drug treatment. Monitoring and acquisition of impedance, resistance and capacitance was performed using ECIS software at multiple AC frequencies. To assess the effect of FFA1 ligands on barrier function, and the paracellular path, the resistance at 4000 Hz frequency averaged for the cells grown on 40 electrodes/well, was normalized to the value before the addition of the compound and plotted as a function of time [43,44,63]. 

### 4.5. Immunofluorescence

Immunofluorescence studies were performed as previously described [63]. RBMVEC grown on 12 mm in diameter fibronectin-coated glass coverslips were treated with AMG837 (10 µM, 30 min) and then processed for immunocytochemistry studies; untreated cells served as controls. Cells were first washed with phosphate buffer saline (PBS) and then fixed with 4% paraformaldehyde (20 min, room temperature). Cells were washed in PBS and PBS with 0.5% Triton X for 5 min and incubated in normal goat serum (1:20, 1 h, room temperature). Cells were incubated overnight at 4 °C with the following primary antibodies: ZO-1 (1:200, rabbit polyclonal, Cat # 40–2200, Thermo Fisher Scientific, Waltham, MA, USA) and VE-Cadherin (1:200, rabbit polyclonal, Cat # 36–1900, Thermo Fisher Scientific) followed by incubation with secondary antibody Alexa Fluor 488 goat anti-rabbit IgG (1:200, Cat # A11008, Thermo Fisher Scientific) or Alexa Fluor 568 goat anti-rabbit IgG (1:200, Cat # A11011, Thermo Fisher Scientific) for 2 h at room temperature. Additionally, cells were incubated in ActinRed 555 (Thermo Fisher Scientific) for 30 min at room temperature. Cells were washed in PBS and then mounted with DAPI Fluoromount G (SouthernBiotech, Birmingham, AL, USA) on glass microscope slides. Cells were examined under a Leica DMI6000B fluorescence microscope equipped with the appropriate filters. 

### 4.6. Evans Blue Extravasation Method

In vivo assessment of BBB permeability was carried out using Evans Blue method, as reported earlier [62,63]. Evans Blue (2% in PBS; 4 mg/Kg, i.v. via tail vein) was administered 30 min before the i.v. injection of FFA1 ligands or vehicle (DMSO, 0.1% *v*/*v*). Two hours or 72 h later, rats were anesthetized with ketamine (100 mg/kg) and xylazine (5 mg/kg) and perfused transcardially with PBS. After dissection, the brain was weighed and homogenized in PBS, then treated with trichloroacetic acid (80%, 1 h, 4 °C). After centrifugation (20 min, 10,000× *g*), the absorbance (610 nm) of the supernatant was determined using a plate reader and the brain concentration of Evans Blue determined.

### 4.7. Assessment of In Vivo BBB Permeability Using Miniaturized Fluorescence Microscopy (Miniscope)

Surgical methods for cannula implantation were as described previously [30,31]. Briefly, rats were anesthetized with isoflurane, and imaging cannula (Miniscope GRIN lens, Doric Lenses, Inc., Quebec, QC, Canada) implanted into the prefrontal cortex using the following stereotaxic coordinates from bregma (AP: 3 mm, ML: 0.5 mm, DV: 2.6 mm) [64]. After two weeks of recovery from surgery and habituation to the study procedures, experiments began. Sodium fluorescein (Na-F; 200 mg/kg, i.v.) was injected through the tail vein followed 15 min later by AMG837 (2 mg/kg, 250 µL) or vehicle. Na-F extravasation, as an indicator of BBB permeability, was measured immediately prior to (i.e., baseline) and again 30 min and 72 h following AMG837 injection. Fluorescence intensity (Ex/Em 488/520 nm) was measured in 10 regions of interest (ROIs) in close proximity of microvessels [30,31]. The same 10 ROIs were recorded prior to and following AMG837 administration using a within-subjects design. Fluorescence was visualized, recorded and analyzed post-acquisition using Doric Studio software (Doric Lenses, Inc., Quebec, QC, Canada).

### 4.8. Statistical Analysis

Data are expressed as the mean ± standard error (SE). One-way ANOVA followed by post hoc analysis using the Bonferroni test was used to evaluate significant differences between groups; two-sample *t*-test was used when comparing two different groups; *p* < 0.05 was considered statistically significant. 

## Data Availability

The data generated and analyzed during this study are available in the manuscript.
